# Microbiological, antioxidant and metabolomics changes in fermented dairy products supplemented with Matzhu

**DOI:** 10.1007/s44463-025-00005-0

**Published:** 2026-02-11

**Authors:** Rui Wang, Malina Kuerman, Chengjie Ma, Kun Wang

**Affiliations:** 1 State Key Laboratory of Dairy Biotechnology, Shanghai Engineering Research Centre of Dairy Biotechnology, Dairy Research Institute, Bright Dairy & Food Co., Ltd, No. 2680, Hechuan Road, Minhang District, Shanghai, 201100 China; 2 Department of Bioengineering, College of Life Sciences and Technology, Xinjiang University, Ürümqi, 830046 China

**Keywords:** Antioxidant activities, Bamboo leaves, Fermented dairy products, *Lacticaseibacillus casei*, Matzhu

## Abstract

Matzhu is a novel natural raw food rich in flavonoids, polysaccharides, and other bioactive compounds; however, its potential applications in the food industry remain largely underexplored. Milk supplemented with 0%, 0.5%, 1%, and 1.5% (w/v) Matzhu was fermented using *Lacticaseibacillus casei* LC2W. The survival of bacteria and the antioxidant activity in the resulting fermented dairy products were assessed during storage and following simulated gastrointestinal digestion in vitro. Notably, the inclusion of Matzhu significantly enhanced the viable counts of *L. casei* LC2W throughout the 21-day storage period, as well as after simulated gastrointestinal digestion. Furthermore, DPPH^+^ scavenging activity of the four groups (L_0_, L_0.5_, L_1_, and L_1.5_) were 20.87%, 31.26%, 36.17%, and 40.94%, respectively. ABTS^+^ scavenging activity also increased, following a similar trend. These results suggest that dairy products treated with Matzhu exhibited markedly increased antioxidant activity compared with those without Matzhu treatment (*p* < 0.05). Untargeted metabolomic analysis identified 1269 differentially expressed metabolites across 14 classes, including unsaturated fatty acids, flavonoids, amino acids, and their derivatives. These metabolites predominantly participate in linoleic acid metabolism, amino acid metabolism, flavonoid and flavonol biosynthesis pathways, and ABC transport mechanisms. Consequently, Matzhu improves the functionality of fermented dairy products by promoting the proliferation of *L. casei* LC2W, enhancing their antioxidant properties, and influencing the production of various beneficial metabolites. In conclusion, integrating Matzhu with probiotics in fermented dairy products presents an exemplary model offering numerous potential health benefits.

**Supplementary Information:**

The online version contains supplementary material available at 10.1007/s44463-025-00005-0.

## Introduction

Fermented foods have a long history worldwide and are becoming popular because of their unique flavours and health benefits. Probiotics are widely used in the production of fermented foods and have numerous health properties including the regulation of intestinal immunity (Kim et al., 2020) and anti-aging (Guarente et al., 2024). One of the key indicators of probiotic performance is their ability to survive during storage. According to the International Dairy Federation recommendations, probiotic products should have a minimum therapeutic standard of lactic acid bacteria of at least 7.0 log CFU/mL (Znamirowska et al., 2021). However, probiotics are highly sensitive to alterations in the external environment, including changes in nutrients and pH. In fermented dairy products, where cow’s milk serves as the primary source of nutrients, insufficiency of carbon and nitrogen sources can readily cause significant attenuation of probiotic activity during the storage period (Fiocco et al., 2020). Therefore, it is of particular significance to adopt measures to facilitate probiotic growth and survival. The development and use of botanical ingredients in the food industry are becoming increasingly widespread, not only because of their diverse flavours, but also because of their rich functional properties. Studies have shown that botanical ingredients have a positive effect on the growth of probiotics because of the presence of various carbohydrates, phenolics, minerals, and other ingredients and have the potential to be used as prebiotics (Mashudin et al., 2024). The discovery of interactions between these components and probiotics during fermentation, as well as their effective incorporation in dairy products, contributes to flavour innovation and improved nutritional value. Previous studies have shown that *Lacticaseibacillus casei* LC2W was found to have beneficial effects on the immune system, intestinal barrier function, hypertension, and metabolic syndrome (Wang et al., 2024a), and has been used in commercial products such as cheese and yoghurt owing to its multiple probiotic properties (Ai et al., 2016; Hong-Xin et al., 2015).

Matzhu (bamboo-leaf superfine powder of *Pleioblastus amarus (Keng) Keng f*.) is an ultrafine powder formed by a series of processes such as blanching and nanosizing of bamboo leaves with reference to the production method of matcha. Matzhu is rich in a variety of nutrients, such as dietary fibre, vitamins, and minerals, in addition to a variety of active ingredients that are beneficial to human health, such as bamboo leaf flavonoids and bamboo polysaccharides (Tundis et al., 2023). The beneficial effects of botanicals are highly contingent upon their inherent bioavailability. Fermentation constitutes an effective technology for enhancing the utilisation of plant-based foods because probiotics can enzymatically degrade some of the active ingredients into more bioavailable compounds through numerous metabolic processes (e.g. de-glycosylation and de-hydroxylation). For example, *Lactobacilli* converted bound phenols in avocado leaf extracts to free phenols via β-glucosidase, thereby increasing their bioavailability (De Montijo-Prieto et al., 2023). Similarly, fermentation of bamboo leaf juice by *Streptococcus thermophilus* for 48 h resulted in significant increases in the polysaccharide, flavonoid, and polyphenol contents of the product, with a 12.1-fold increase in the polysaccharide content (Fei et al., 2023). However, the development and utilisation of bamboo leaf resources are still insufficient, and the main focus in the food industry is on the application of bamboo leaf flavonoids and polysaccharides (Hu et al., 2023). Although Matzhu is an emerging food ingredient, studies on the growth and metabolic status of its probiotics have not yet been conducted and their application in fermented foods is limited.

Current fermented dairy products are mostly made with botanical ingredients added after the completion of fermentation, however, the addition of these ingredients during the fermentation process may improve the final product by influencing bacterial growth and metabolism. Considering the potential prebiotic role of Matzhu, this study aimed to evaluate its effect milk fermented with *L. casei* LC2W, specifically the survival of the bacteria and antioxidant capacity of the fermented milk product, assessed following storage and after simulated in vitro digestion of the product. This study was also designed to evaluate the changes in non-volatile metabolites in fermented dairy products and the health benefits associated with the addition of Matzhu. The results provide a theoretical foundation for future research on the application of Matzhu in fermented dairy products.

## Materials and methods

### Materials

The nutrient contents of raw milk (sourced from Shanghai Bright Dairy Co., Ltd) and Matzhu (purchased from Sichuan Senlong Biological Co., Ltd) are presented in Tables A.1 and A.2 (supplementary material). The lyophilised probiotic cultures of *L. casei* LC2W (Shanghai Bright Dairy Co., Ltd) (Zhang et al., 2021) were activated at 37 °C in 10% (w/v) sterile reconstituted milk for 24 h, and 1% (*v*/*v*) of the culture was inoculated again for reactivation under the same conditions.

### Water solubility index and water holding capacity of Matzhu

The water solubility index (WSI) and water holding capacity (WHC) were determined using the gravimetric method described by Najman et al. (2023). Briefly 2.5 g (accurate to 0.0001 g) of Mazhu was weighed into a 50 mL centrifuge tube and dissolved in 30 mL of distilled water with thorough agitation (37 ± 1 °C, 30 min) and then centrifuged at 640×*g* for 20 min. The supernatant was collected and the weight of the precipitate was recorded. The supernatant was dried to constant weight in an incubator (103 ± 2 °C) and weighed. WHC and WSI were measured using the following formulae:

1 $$WHC\left( \% \right) = \left( {W_{1} /W_{0} } \right) \times 100$$

2 $$WSI\left( \% \right) = \left( {W_{3} /W_{2} } \right) \times 100$$

W_0_ (g) is the weight of the sample before centrifugation, W_1_ (g) is the weight of the de-watered sample after centrifugation, W_2_ (g) is the weight of the supernatant after centrifugation, and W_3_ (g) is the weight of the supernatant after it has been dried to constant weight.

### Formulation and storage of the fermented dairy product

After the standardized raw milk was heated to 65 °C, homogenization was performed at a pressure of 250 bar. Subsequently, Matzhu was added to the homogenized milk at concentrations of 0.5%, 1%, and 1.5% (w/v), followed by thorough mixing. The amount of Matzhu added was determined based on a study by Fu et al. (2019). Control samples were prepared without Matzhu. The mixtures were sterilized at 95 °C for 10 min and subsequently cooled to 42 °C for storage. Each sample was then inoculated at 1% (*v*/*v*) with *L. casei* LC2W (concentration of 9.0 log CFU/mL) and fermented until the pH decreased to 4.6 ± 0.1 (Plessas et al., 2024), monitored using a pH meter. The pH and total bacterial counts were analysed on days 1, 7, 14 and 21 of refrigeration (4 ± 2 °C). To estimate the number of colonies in the samples, serial dilutions were performed, and inoculations onto MRS agar medium plates were carried out. The plates were then incubated in a constant-temperature incubator at 37 ± 0.2 °C for 48 h, and the number of colonies were counted (Alvarado et al., 2024).

### Antioxidant capacity

#### α,α-Diphenyl-β-picrylhydrazyl assay

The antioxidant capacities of different samples were assessed using the α,α-diphenyl-β-picrylhydrazyl (DPPH) method as described in previous studies, with minor modifications (De Montijo-Prieto et al., 2023). Briefly, the DPPH stock solution was prepared by dissolving 0.4 g (accurate to 0.0001 g) of DPPH powder in anhydrous ethanol (100 mL). This stock solution was subsequently diluted tenfold. A volume of 3.9 mL of the diluted solution was transferred to a 5 mL centrifuge tube, followed by the addition of 0.1 mL of the sample. The mixture was incubated in the dark for 30 min and centrifuged at 640×*g* and 4 °C for 5 min. The absorbance of the supernatant was measured at 517 nm using a spectrophotometer (Genesis 10 s; Thermo Scientific, USA). Antioxidant activity was measured using the following formula:

3 $$DPPH^{ + } \;Scavenging \; activity\left( \% \right) = \frac{{A_{0} - A}}{{A_{0} }} \times 100$$

A_0_ and A are the absorbance of the control and sample respectively.

#### 2,2′-Azino-bis (3-ethylbenzothiazoline-6-sulfonic acid) assay

A sample (10 g; accurate to 0.0001 g) was centrifuged at 640×*g* and 4 °C for 5 min. The resulting supernatant was subsequently filtered through a 0.22 μm membrane and stored in a refrigerator at 4 °C. In accordance with the manufacturer’s guidelines, 2,2′-azino-bis (3-ethylbenzothiazoline-6-sulfonic acid) (ABST) solution and oxidant solution from the reagent kit were prepared in equal proportions of the ABST masterbatch and allowed to incubate at room temperature for 16 h in a dark environment. For the assay, 280 μL of a 50-fold dilution of ABST master mix was combined with 7 μL of the sample supernatant and incubated at room temperature for 5 min, and the absorbance was measured at 405 nm. Antioxidant activity was measured using the following formula:

4 $$ABST^{ + } \;Scavenging \;activity\left( \% \right) = \frac{{B_{0} - B}}{{B_{0} }} \times 100$$

B_0_ and B are the absorbance of the control and sample respectively.

### In vitro simulation of gastrointestinal conditions

In vitro simulation of gastrointestinal digestion was carried out according to the method described by Alvarado et al. (2024), with minor adjustments. Briefly, the prepared saliva, intestinal, and gastric fluids were adjusted to a specific pH and then filtered through a sterile 0.22-μm syringe. The samples were warmed to 37 °C before use. The oral, intestinal, and gastric environments were sequentially simulated, and the digested solution was removed at the end of each digestion phase and placed on ice to quench enzyme activity, and thereafter the antioxidant activity and viable bacterial counts were determined. The digestate-to-solution volume ratio was maintained at 1:1 throughout the experiment.

### Untargeted metabolomics

Non-targeted metabolomic analyses were performed using a tandem Fourier transform mass spectrometry UHPLC-Q Exactive system (Thermo Fisher, Palo Alto, CA, USA), in accordance with the method described by Ren et al. Chromatographic separation was carried out on a HSS T3 column (100 mm × 2.1 mm i.d., 1.8 µm) at 40 °C. Mobile phase A was 95% water + 5% acetonitrile (containing 0.1% formic acid) and mobile phase B was 47.5% acetonitrile, 47.5% isopropanol, and 5% water (containing 0.1% formic acid). Mass spectrometry signals were obtained in positive and negative ion scanning modes with a mass scanning range of 70–1050 *m*/*z* and a resolution of 70,000. Raw LC–MS data were imported into the metabolomics processing software Progenesis QI (Waters Corporation, Milford, USA) for in-depth analysis, whereas MS and MS/MS mass spectra were transferred to the human metabolome database (HMDB; http://www.hmdb.ca/) and Metlin (https://metlin.scripps.edu/). Principal component analysis (PCA) of metabolites was performed to comprehend the distinctions between groups of samples and the magnitude of variability between samples within groups. By optimizing the differences between groups, orthogonal partial least square discriminant analysis (OPLS-DA) provides a better separation than PCA. Metabolite identification was based on the HMDB and Kyoto Encyclopedia of Genes and Genomes (KEGG; https://www.genome.jp/kegg/) databases. All analyses were performed using Meggie BioCloud (https://cloud.majorbio.com).

### Statistical analysis

The results are expressed as mean ± standard deviation (SD) of three independent determinations unless otherwise stated. Statistically significant differences between samples were determined by analysis of variance (ANOVA) followed by Tukey’s post-hoc test.

## Results

### Characteristic of Matzhu

**Fig. 1:**
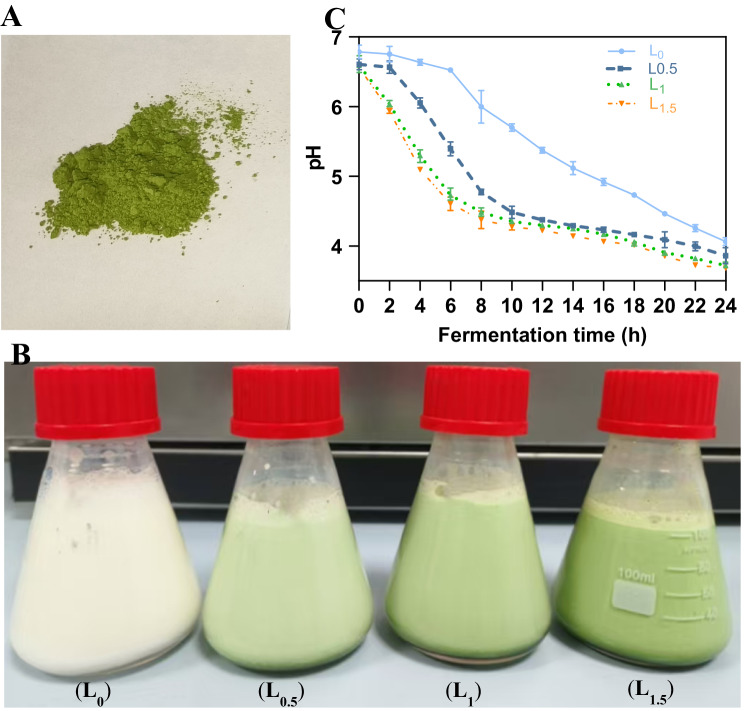
The photograph of **A** Matzhu powder; **B** fermented dairy products: 0, 0.5, 1, and 1.5% (w/v) Matzhu; Changes in pH (**C**) and viable cell counts (**D**) during the fermentation of fermented dairy products supplemented with 0–1.5% (w/v) Matzhu. L_0_, L_0.5_, L_1_ and L_1.5_ represent Matzhu supplemented with 0%, 0.5%, 1%, and 1.5% (w/v), respectively. Upper case letters indicate inter-group variability at the same time, with A–D marking a significant difference among different superscript letters (*p* < 0.05); lower case letters represent intra-group variability at different times, with a–c marking a significant difference among different superscript letters (*p* < 0.05)

Matzhu and its milk state are shown in Fig. 1A and B, respectively, while its basic properties are summarised in Tables A.1 and A.2 (supplementary material). Matzhu appeared loose, dry, and free of lumps with the drink’s colour deepening as the Matzhu content increased. WSI and WHC are the core functional indicators of Matzhu applications, as they directly influence the organoleptic properties, functional value, and final product quality. A thorough understanding of these two indicators can guide subsequent research to enhance them, thereby expanding the potential of Matzhu applications while simultaneously improving production efficiency and consumer experience. The results showed that Matzhu had a WSI of 12.225 ± 0.465% and a WHC of 35.272 ± 1.561% (Table 1). The changes in pH during fermentation are shown in Fig. 1C. The pH decreased to 4.6 ± 0.1 at approximately 20 h of fermentation for L_0_, at approximately 12 h for L_0.5_, and at approximately 8 h for L_1_ and L_1.5_, demonstrating that Matzhu accelerated the pH decline and shortened the fermentation time. The faster decrease in pH with the addition of Matzhu may be attributed to the biodegradation effect that may result in a more complete release of organic acids from Matzhu, and on the other hand, Matzhu provides more nutrient-rich components thus allowing faster microbial growth, which accelerates the rate of acid production. As shown in Fig. 1D, at the end of fermentation (24 h), the samples supplemented with 1% and 1.5% Matzhu have grown to 12.21 log CFU/mL and 12.19 log CFU/mL, which were greater than L_0_ and L_0.5_ (9.37 and 9.81 log CFU/mL). Moreover, the addition of Matzhu to the milk significantly increased the viable cell counts of *L. casei* LC2W when the fermentation time exceeded 4 h (*p* < 0.05), suggesting that Matzhu promoted the growth of *L. casei* LC2W. In lactic acid bacteria, the pH decrease correlates with growth properties. Substances, such as polysaccharides can stimulate bifidobacterial growth and enhance metabolic processes promoting acid production (Guan et al., 2025). Therefore, we hypothesized that Matzhu positively influences the metabolic activity and growth of *L. casei* LC2W.

**Table 1:** The detection of WSI and WHC in Matzhu

Element	WSI (%)	WHC (%)
Matzhu	12.225 ± 0.465	35.272 ± 1.561

Both indicators were subjected to 3 replications of the experiment. All values are shown as mean ± SD

### Microbiological and antioxidant activities of a fermented dairy product during storage

**Fig. 2:**
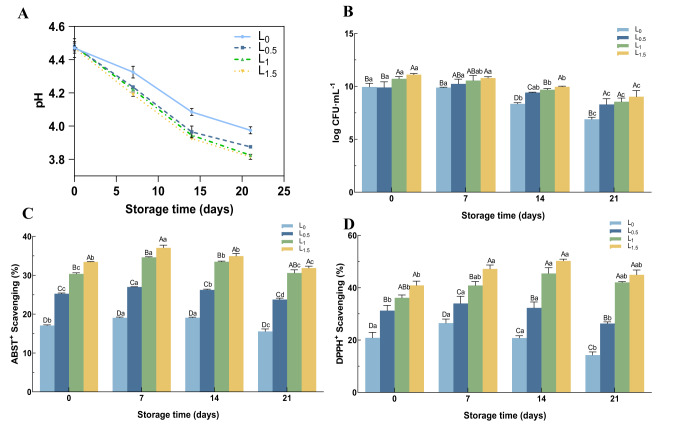
pH values (**A**), viable cell counts (**B**), the ABTS radical scavenging capacity of the beverages (**C**) and the DPPH radical scavenging capacity (**D**) in the fermented dairy products using *L. casei* LC2W during 21-day storage period. Upper case letters indicate inter-group variability at the same time, with A–D marking a significant difference among different superscript letters (*p* < 0.05); lower case letters represent intra-group variability at different times, with a–c marking a significant difference among different superscript letters (*p* < 0.05)

The four groups of samples were fermented to pH 4.6 ± 0.1 and stored 4 °C. They were tested for pH, viable counts, and antioxidant properties at 7-day intervals over a 21-day storage period. As shown in Fig. 2A, at the end of the storage period, the pH reached 3.92 in L_0_, 3.83 in L_0.5_, 3.77 in L_1_, and 3.75 in L_1.5_. Thus, the addition of Matzhu accelerated the reduction in pH to a certain extent, suggesting that *L. casei* LC2W may have a stronger metabolic activity and even generate organic acids during refrigerated storage, thereby lowering the pH. As shown in Fig. 2B, viable cell counts in all groups declined over time during storage, which was associated with a decline in pH and a reduction in metabolizable nutrients. However, the viable cell counts of all three groups supplemented with Matzhu were above 7.0 log CFU/mL during refrigeration. By the end of storage, the viable cell counts in the Matzhu containing groups were significantly higher than those in L_0_. These results indicate that Matzhu promotes the growth of *L. casei* LC2W and enhances strain survival during the storage period. As shown in Fig. 2, DPPH^+^ and ABTS^+^ scavenging activities demonstrated a concentration-dependent relationship with Matzhu concentration, showing a gradual increase as the content of Matzhu increased. Thus, the formulation with 1.5% Matzhu had the highest antioxidant activity, with mean scavenging activities of 45.86% and 34.35% for DPPH^+^ and ABST^+^, respectively, throughout the storage period (Fig. 2C, D), which were significantly higher than those of L_0_ (*p* < 0.05). Overall, the average scavenging rates of L_1_ throughout the storage period were 41.14% and 32.27% for DPPH^+^ and ABST^+^ scavenging activities, respectively (Fig. 2C, D), and no significant difference was observed in the antioxidant activity during the later stages of storage as compared to L_1.5_ (*p* > 0.05).

### Survival of *L. casei* LC2W and the antioxidant activity of fermented dairy products subjected to in vitro gastrointestinal digestion

**Fig. 3:**
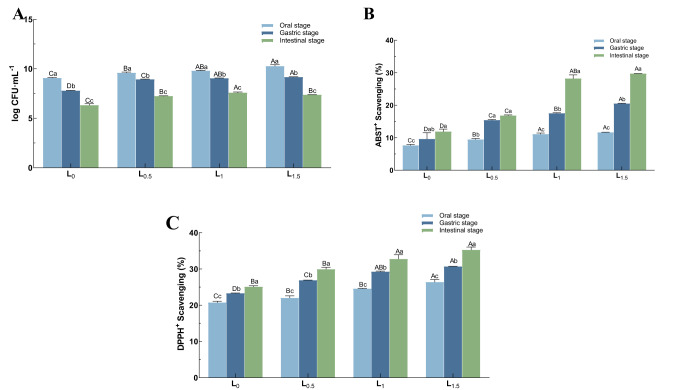
Viable cell counts (**A**), determination of the ABTS (**B**) and DPPH (**C**) radical scavenging capacity of the fermented dairy products after simulation of the gastrointestinal digestion. Upper case letters indicate inter-group variability at the same time, with A–D marking a significant difference among different superscript letters (*p* < 0.05); lower case letters represent intra-group variability at different times, with a–c marking a significant difference among different superscript letters (*p* < 0.05)

The survival of *L. casei* LC2W during in vitro digestion is illustrated in Fig. 3A. Cell numbers of *L. casei* LC2W in L_0.5_, L_1_, and L_1.5_ were respectively, 9.25, 9.85 and 10.41 log CFU/mL for oral digestion; 8.91, 9.07 and 9.19 log CFU/mL for gastric digestion; and 7.23, 7.53 and 7.39 log CFU/mL for intestinal digestion. After gastrointestinal simulated digestion, viable cell counts were significantly lower in all groups (*p* < 0.05). Compared to L_0_, Matzhu-containing samples had higher viable cell counts at all stages, and at the end of the last digestion, the microbial survival was higher in L_1_ and L_1.5_ (*p* < 0.05 versus L_0_ for each), with mean values of 7.53 and 7.39 log CFU/mL, respectively. The results indicate that dairy products fermented with Matzhu maintained better strain viability and stability during digestion.

As depicted in Fig. 3A and B, antioxidant activities across all groups increased to varying degrees through digestion; L_0.5_, L_1_, and L_1.5_ exhibited greater increases than L_0_, which may be due to digestive enzyme action enhancing the release of phenolics, flavonoids and their derivatives from Matzhu, and an increase in free amino acids and peptides released from the milk protein (Leonard et al., 2021). This was particularly prominent in L_1_ and L_1.5_, which did not differ significantly in antioxidant activity after intestinal digestion.

### Metabolomics analysis of fermented dairy products supplemented with Matzhu

#### Principal component analysis and orthogonal partial least square discriminant analysis of metabolites

**Fig. 4:**
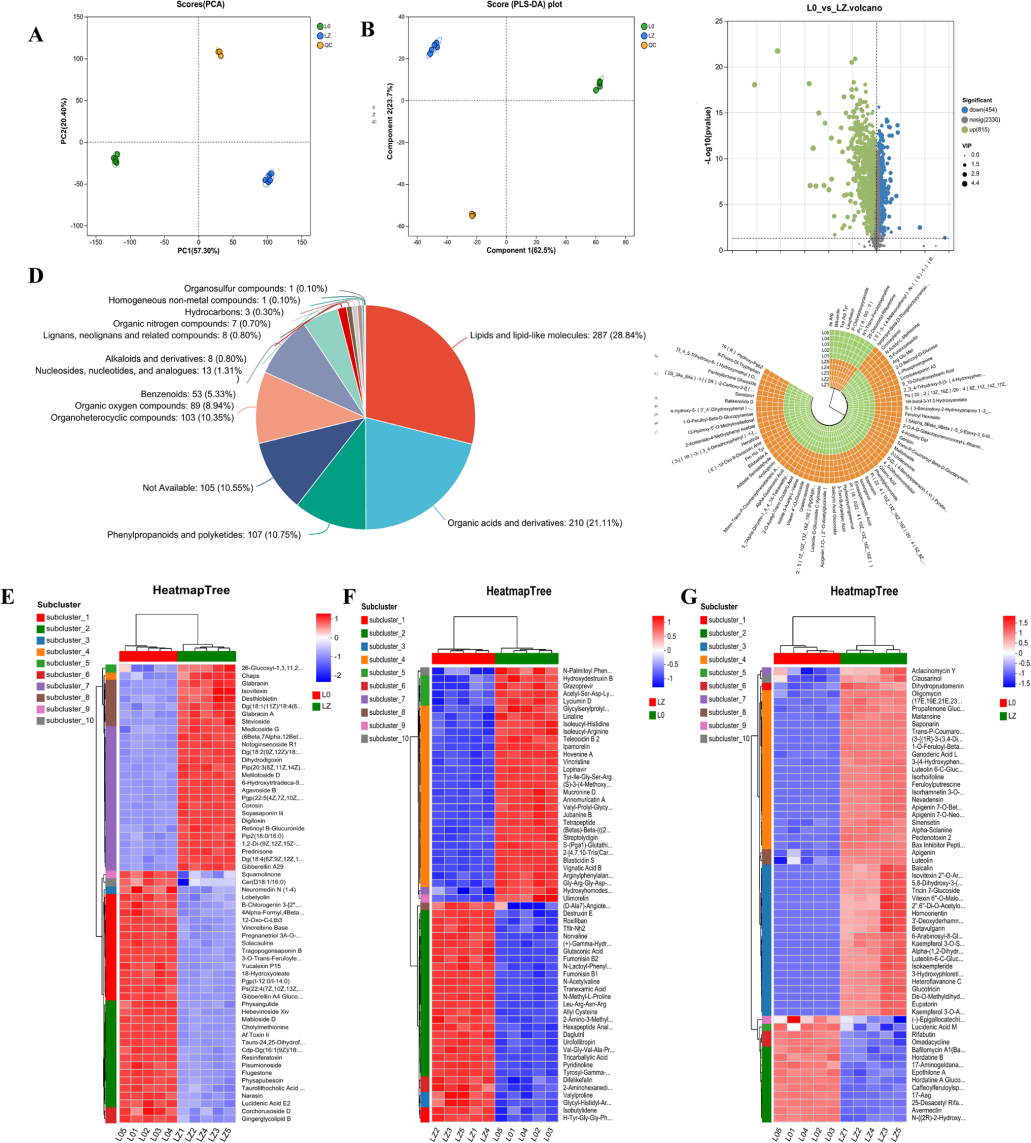
PCA analysis (**A**); permutation plot test of OPLS-DA model (**B**); differential volcano map (**C**); classification analysis of HMDB compounds (**D**); Heatmap of hierarchical clustering analysis in lipid and lipid-like molecules (**E**), category of organic acids and derivatives (**F**), phenylpropanoids and polyketides (**G**)

Both L_1_ and L_1.5_ demonstrated superior performance in terms of antioxidant activity and probiotic protection, with only a marginal difference between them. Therefore, considering resource conservation in the actual production process, L_1_ (denoted hereafter as LZ) was selected for subsequent analyses, L_0_ (denoted hereafter as L0) was used as the blank control group. We used non-targeted metabolomics analysis to study the metabolite changes between L0 and LZ. The metabolites were subjected to PCA (Fig. 4A) and OPLS-DA (Fig. 4B). As shown in Fig. 4, the quality control (QC) samples were well grouped, indicating good bioanalysis and data quality. This means that group separation was due to variables rather than the analysis process. The LC–MS samples in the positive and negative modes were in the confidence circle (95% confidence level), and PC1 and PC2 accounted for 57.30% and 20.40% of the total variance, respectively, thus allowing for sample separation by PCA. OPLS-DA showed that components 1 and 2 explained 62.5% and 23.7% of the variance, respectively. The significant classification effect between the groups indicated a well-fitted predictive model suitable for subsequent data analysis.

#### Identification and analysis of differential metabolites in fermented dairy products supplemented with Matzhu

Using the KEGG database, we identified 1269 metabolites that differed significantly from the control group based on the criteria of VIP > 1.5 (OPLS-DA model), |log_2_(FC)| > 1, and FDR-adjusted *p* < 0.05. Among these metabolites, 815 were up-regulated and 454 were down-regulated (Fig. 4C). As shown in Fig. 4D, the metabolites were classified into the following 14 compound classes: lipids and lipid-like molecules; organic acids and derivatives; phenylacetones and polyketides; others; organic heterocyclic compounds; organic oxygenated compounds; benzene analogues; nucleosides, nucleotides, and analogues; alkaloids and their derivatives; lignans, neo-lignans, and related compounds; organic nitrogen compounds; hydrocarbons; homogeneous non-metallic compounds; and organosulfur compounds. Notably, lipid and lipid-like molecules, organic acids and derivatives, and phenylpropanoids and polyketides comprised the largest proportion (60.70%) of the total metabolites. The top 60 significant metabolites in these categories were clustered for analysis.

Among lipid and lipid-like molecules, compared to L0, LZ showed significant decrease and increases in 33 and 27 metabolites, respectively (Fig. 4E). Similarly, among organic acids and derivatives, LZ showed a significant decrease in 31 metabolites, whereas 29 metabolites were increased-mainly amino acids and derivatives linked to antioxidant effects observed in LZ (Fig. 4F). Among the phenylpropanoids and polyketides, there were 14 downregulated and 46 upregulated metabolites in LZ (Fig. 4G). Cluster analysis revealed that nine of the top 74 metabolites decreased in LZ, whereas others increased (Fig. 4H), including 17 lipid and lipid-like molecules, 10 polyketides, 8 organic oxygenates, 7 organic heterocyclic compounds, and 5 organic acids and their derivatives. Of the 1269 differential metabolites, those with high contribution to the classification were screened based on VIP and *p* values, and 20 metabolites closely related to antioxidant activity were screened from these metabolites using pathway analysis; these included unsaturated fatty acids, saponins, lignans, flavonoids and their derivatives, as well as phenolic acids and their derivatives (Table 2). Among these 20 metabolites, flavonoids and phenolic acids were the most abundant. The flavonoids included vitexin 4′-*O*-glucoside, kaempferol 3-*O*-sophoroside, isovitexin, apigenin, galangin, luteolin, and naringenin. The phenolic acids included isoferulic acid, 2-hydroxycinnamic acid, protocatechuic acid, and 3-hydroxybenzoic acid. Additionally, lignans (e.g., syringaresinol, 2-hydroxyarctiin), terpenoids (e.g., licoricesaponin A3, acuminoside, bakkenolide D), fatty acids, and other active ingredients (e.g., 2-Hydroxymyristic Acid, Linoleic Acid, Melilotoside) were also identified. According to extensive literature reviews, substances with antioxidant activity predominantly consist of natural compounds such as phenols (Wu et al., 2021), flavonoids (Chen et al., 2022), and terpenoids (Yu et al., 2024). Their mechanisms of action involve free radical scavenging, modulation of oxidative stress, and activation of signalling pathways (Adhikari & Saha, 2024; Tundis et al., 2023). Compared to L0, these metabolic components were markedly upregulated in LZ (Table 2), and contributed to the substantial enhancement of antioxidant properties.

**Table 2:** Key metabolites in fermented dairy products supplemented with Matzhu

Metabolite	Abundance		*p*_value	VIP_pred_OPLS-DA	FC	log_2_FC	FDR	Significant
	L0	LZ						
Bakkenolide D	2.892	5.585	4.82E−19	2.8313338	0.5178156	−0.94949	1.80E−15	Yes
Melilotoside	3.991	5.244	2.19E−16	1.9313392	0.7610603	−0.393917	9.72E−14	Yes
Syringaresinol	1.751	4.858	4.43E−14	3.0402601	0.3604364	−1.472183	6.09E−12	Yes
Licoricesaponin A3	3.913	5.717	4.58E−14	2.3171364	0.6844499	−0.546983	6.18E−12	Yes
Vitexin 4″-*O*-glucoside	3.585	6.302	9.59E−14	2.8430581	0.568867	−0.813837	1.09E−11	Yes
Acuminoside	3.948	5.368	5.93E−13	2.0552614	0.7354694	−0.443263	4.42E−11	Yes
Isoferulic acid	3.388	4.823	2.45E−12	2.0653395	0.7024673	−0.509497	1.33E−10	Yes
Luteone	2.898	5.047	1.00E−11	2.5272433	0.5742025	−0.800368	3.98E−10	Yes
Naringenin	2.682	4.902	2.67E−11	2.5684371	0.5471236	−0.870061	7.43E−10	Yes
2-Hydroxyarctiin	4.271	5.176	7.61E−11	1.6389133	0.8251546	−0.277264	9.49E−10	Yes
2-Hydroxycinnamic acid	4.458	5.006	3.61E−10	1.2755773	0.8905314	−0.167262	3.68E−09	Yes
Protocatechuic acid	4.555	5.063	3.23E−07	1.2190801	0.8996642	−0.152541	1.35E−06	Yes
Kaempferol 3-*O*-sophoroside	4.937	5.585	2.02E−08	1.3832235	0.8839749	−0.177923	1.23E−07	Yes
3-Hydroxybenzoic acid	5.618	5.977	3.37E−08	1.027744	0.9399364	−0.089365	1.92E−07	Yes
Isovitexin	6.967	7.509	1.39E−07	1.2617414	0.9278199	−0.108083	6.51E−07	Yes
Apigenin	3.153	6.654	7.65E−07	3.1940412	0.4738503	−1.077497	2.566E−06	Yes
Galangin	3.032	4.117	8.12E−07	1.7782623	0.7364586	−0.441324	2.706E−06	Yes
2-Hydroxymyristic acid	5.425	5.951	1.388E−06	1.2364179	0.9116115	−0.133509	4.345E−06	Yes
Linoleic acid	4.436	6.544	1.705E−06	2.4729297	0.6778729	−0.560913	5.206E−06	Yes
Luteolin	3.604	6.066	0.00006942	2.617361	0.5941312	−0.751146	0.0001444	Yes

(1) Metabolite: the name of the identified metabolite; (2) Abundance: the relative expression level of the metabolite in each sample group; (3) *p* value: the statistical significance of the difference in expression levels of this metabolite between the two sample groups, with a default screening criterion of VIP_pred_OPLS-DA > 1 and *p* value <0.05; (4) VIP_pred_OPLS-DA: the VIP value indicates the strength of the effect of between-group differences in the categorical discrimination of each group of samples in the model for the corresponding metabolite, and a metabolite with a VIP ≥ 1 is generally considered to be significantly different; (5) FC: a fold change of 1, i.e., metabolites differing by a factor of 1 or more in the control and experimental groups, is considered significant; (6) FDR: the False Discovery Rate-corrected *p* value

#### Analysis of metabolic pathways involving differential metabolites in fermented dairy products supplemented with Matzhu

**Fig. 5:**
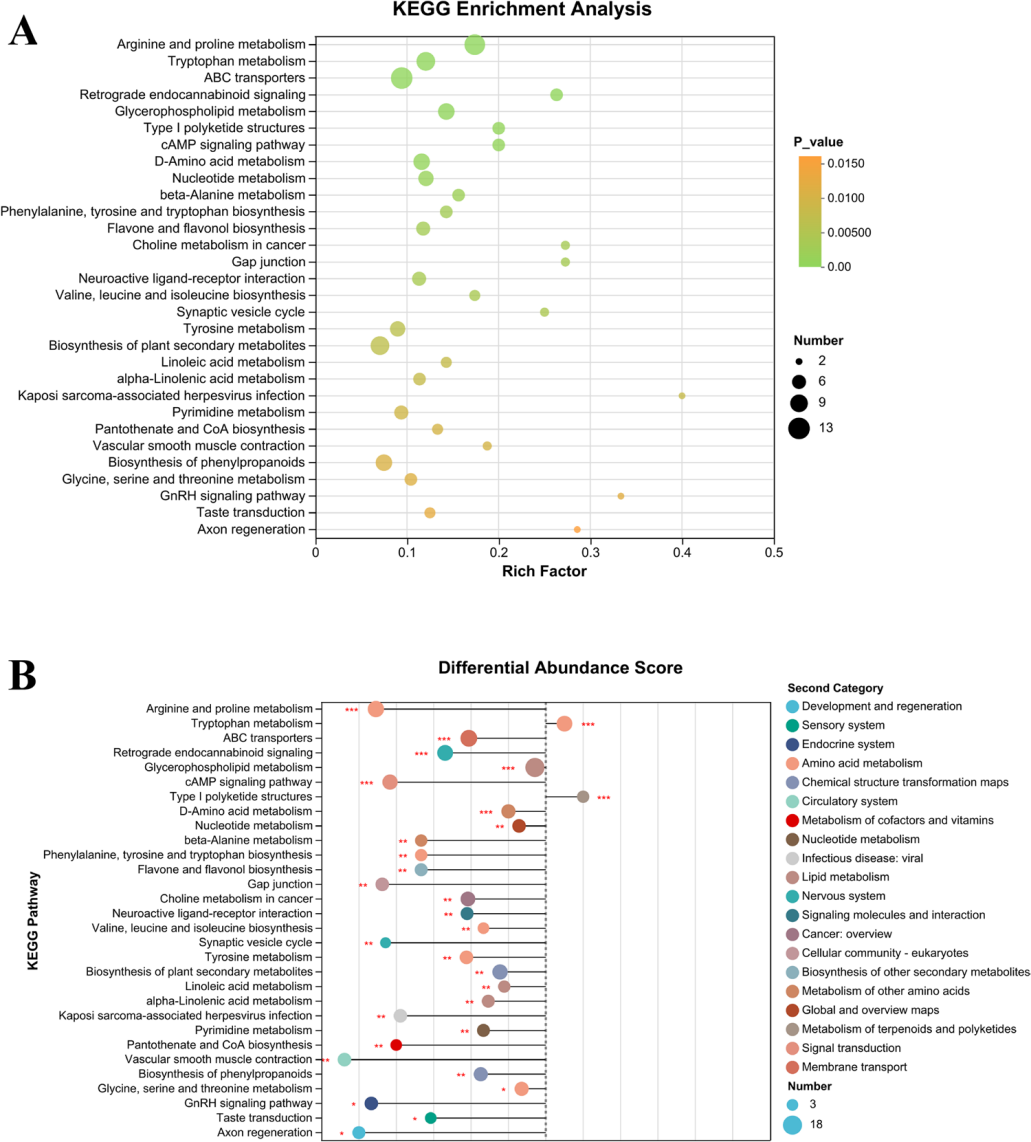
Bubble map of the metabolic pathways based on the differential metabolites (**A**); differential abundance score of identified metabolic pathways (**B**)

KEGG pathway enrichment analysis of 1269 differential metabolites identified a total of 238 metabolic pathways, from which the top 30 pathways ranked by significance were selected (Fig. 5A). Among these pathways, carbohydrate metabolism, fatty acid metabolism, amino acid metabolism, and others are directly related to the metabolic activities of the probiotics. These include processes such as ABC transporter-mediated transport, linoleic acid and α-linoleic acid metabolism, arginine and proline metabolism, D-amino acid metabolism, tryptophan metabolism, and phenylalanine biosynthesis. During probiotic fermentation, microorganisms utilize sugars and fatty acids via these pathways to generate energy and produce metabolites essential for microbial growth (Tang et al., 2023). Moreover, certain metabolic pathways are involved in the biosynthesis of antioxidant components, such as flavonoids and flavonols. As illustrated in Fig. 5B, 28 metabolic pathways were significantly upregulated in the LZ group, whereas tyrosine metabolism and type I polyketide structure were notably downregulated.

## Discussion

In this study, *L. casei* LC2W was used to ferment milk supplemented with 0%, 0.5%, 1%, and 1.5% (w/v) Matzhu. The fermentation endpoint was reached more rapidly in the groups supplemented with Matzhu, and correlated with the concentration of Matzhu added. During storage, pH reduction was more pronounced in samples L_0.5_, L_1_, and L_1.5_ than in L_0_. Previous research indicated that fermented dairy products enriched with taro leaf and moringa extracts exhibited similar pH changes during storage owing to the enhanced metabolic activity of probiotic bacteria leading to organic acid production (Shehata et al., 2023; Zhang et al., 2019). It has been established that at the end of the shelf life, probiotic concentrations must be maintained at a minimum of 7.0 log CFU/mL to ensure that adequate levels survive the adverse conditions within the gastrointestinal tract in order to convey health benefits (Palencia-Argel et al., 2024). The inclusion of Matzhu preserved high cell viability in the fermented dairy products over a period of cold storage of up to 21 days, achieving probiotic concentrations exceeding 7.0 log CFU/mL in the L_0.5_, L_1_, and L_1.5_ formulations. Furthermore, in vitro gastrointestinal digestion assessments revealed higher viable counts for samples L_0.5_, L_1_, and L_1.5_ relative to the control sample L_0_; this protective effect may be ascribed to the substantial fibre content of Matzhu which remained undigested throughout gastrointestinal transit acting as a barrier against digestive enzymes, which is consistent with the investigation of Buriti et al. (2010) into the survival of *L. acidophilus* in guava mousse after digestion. However, the complexity of the gastrointestinal tract environment may attenuate this protective effect. Additionally, the structural and solubility differences among various dietary fibres might offer varying levels of protection to different strains. The synergistic effect between specific dietary fibres and probiotic strains requires further verification in subsequent studies. Notably, all fermented dairy products containing Matzhu demonstrated significant increases in antioxidant properties after simulated gastrointestinal digestion; this is consistent with previous studies indicating that plant extract additions, such as oatmeal and pomegranate peel enhance product antioxidant activities (Al-Hindi & Abd El Ghani, 2020; Cho et al., 2024). Moreover, beyond their inherent strain-specific antioxidant capabilities, certain polyphenols and organic acids are also closely linked to enhanced antioxidant activity (Santos Pereira et al., 2024). Therefore, a comprehensive analysis focusing on relevant metabolite components is warranted to evaluate alterations observed in fermented dairy products supplemented with Matzhu.

Based on these findings, fermented dairy products with 0% and 1% Matzhu content, designated as L0 and LZ, respectively, were selected for non-targeted metabolomics analysis. The results from the PCA and OPLS-DA revealed a distinct segregation trend between L0 and LZ, indicating significant differences in metabolites between the two groups; thus, subsequent differential metabolite analyses were conducted. Through the HMDB compound classification of the differential metabolites, 1269 metabolites across 14 classes were identified. Notably, three categories accounted for the highest proportions of these metabolites: lipid and lipid-like molecules, organic acids and derivatives, and phenylpropanoids and polyketides. Among lipids and lipid-like molecules, unsaturated fatty acids abundantly up-regulated in LZ, such as eicosapentaenoic and linoleic acids, are recognised for their beneficial anti-inflammatory properties (Raman et al., 2017). Flavonoids predominantly exist in glycoside forms which are hydrolysed by cellulases (e.g., β-glucosidase) secreted by microorganisms during fermentation to yield isoflavone glycosides (Curiel et al., 2024). Additionally, ginsenosides, glycyrrhizin A3, and spiny saponins were significantly upregulated in LZ, and these metabolites possessed enhanced antioxidant capabilities (Yu et al., 2024). Organic acids and their derivatives not only enhance the flavour profile of fermented foods but also inhibit unwanted bacterial growth during fermentation processes. Among phenylketones and polyketides, there were 14 downregulated and 46 upregulated metabolites in LZ; notably more flavonoids and other aromatic compounds (e.g., bicoumarins and 7-methoxycoumarins) showed up-regulation than down-regulation; these compounds are primarily acknowledged for their antioxidant activity, but are also known for antiparasitic and antiviral effects. Due to their complex structures, which often hinder absorption within biological systems, flavonoids can undergo structural modifications during microbial fermentation, thereby enhancing their bioavailability. Additionally, they may function as prebiotics, promoting beneficial microorganism proliferation (Chen et al., 2022).

We identified 20 metabolites with significant antioxidant properties among the differential metabolites, including isovitexin, an active compound found in all bamboo products, which exhibited notable hypoglycaemic effects in addition to its antioxidant properties (Yan et al., 2024). Apigenin, widely distributed across various plant species, has been shown to confers multiple protective benefits, including blood pressure reduction and anti-inflammatory effects (Adhikari & Saha, 2024). Phenolic acids, secondary metabolites classified as polyphenols, can be further categorised into esterified and bound phenolic acids; free phenolic acids, of which isoferulic acid is the most abundant, demonstrated superior antioxidant activity (Olech et al., 2020).

Numerous studies have demonstrated that flavonoids and phenolic acids can serve as nutritional substrates for probiotics by supplying them with essential energy and nutrients to facilitate growth and reproduction (Peng et al., 2020). A previous investigation revealed that pomegranate peel extract, which is abundant in phenolic compounds, inhibited the proliferation of *Escherichia. coli*, while simultaneously promoting the growth of probiotics such as *L. plantarum* and *Bifidobacterium bifidum* (Al-Moghazy et al., 2023). Furthermore, mangosteen extracts, rich in phenolic acids and flavonoids at a concentration of 5%, exhibited beneficial effects on both intestinal and commercial probiotic strains, particularly by enhancing their antioxidant and antimicrobial properties (Rodriguez-Minguez et al., 2024). In conclusion, *Lactobacilli* have various enzymes capable of metabolizing flavonoids and phenolic acids, thereby augmenting antioxidant activity (Wang et al., 2024b). Moreover, supplementation with food substrates high in these compounds positively influences *Lactobacillus* growth and functionality (Plamada & Vodnar, 2022). While the transformation of flavonoids and phenolic acids by *Lactobacillus* has been extensively studied, significant knowledge gaps remain regarding the influence of these components on probiotic physiology and function. Thus, further data is required to elucidate the interaction patterns between different strains and components derived from diverse plant sources.

The flavour compounds prevalent in fermented foods primarily arise from the microbial catabolism of nutrients such as proteins, fats, and lactose leading to complex substances such as acids, esters, alcohols, ketones, and aldehydes. Our findings indicated that the differential metabolic pathways between L0 and LZ were chiefly associated with fatty acid and amino acid metabolism, including linoleic acid, arginine, and proline, and glycerophospholipid metabolism. Notably, we observed an increasing trend in relative abundance of these amino acids and their derivatives in LZ. The interplay between amino acid and fatty acid metabolism significantly influences both flavour profile and nutritional value of fermented foods; for instance, unsaturated fatty acid metabolism yields a variety of flavourful ketones and aldehydes, while specific amino acids contribute to freshness or sweetness and support immune functions within organisms (Chi et al., 2024; Zhang et al., 2024). We found a substantial enrichment within the ABC transporter pathway, in which the expression levels of all annotated differential metabolites were markedly up-regulated. The uptake of oligosaccharides by probiotics via ABC transporters activity facilitates their proliferation (Jana et al., 2021), suggesting that Matzhu supplementation may promote the growth of *L. casei* LC2W partly by activating this transporter system. Matzhu enriched the nutrient profile of the fermented dairy products. Metabolic pathways such as those associated with amino acids and fatty acids are better able to provide energy to the strain, which may be beneficial in promoting the growth of the strain. Thus, the addition of Matzhu not only enhances the nutritional properties and flavour characteristics of fermented milk but also has a positive impact on probiotic growth. However, the specific substances and pathways through which this positive effect occur requires further investigation, which may lead to better utilization Matzhu’s prebiotic potential. Additionally, the transformation and degradation mechanisms of the complex chemical components in Matzhu during fermentation, as well as the properties and safety of its fermented products, remain under-investigated, thereby limiting the application potential of Matzhu in fermented foods. Based on the metabolomics results, future studies could systematically investigate the interaction modes (e.g., hydrogen bonding, hydrophobic interactions) between key antioxidant components in Matzhu and milk. The structural characteristics of the resulting complexes could be analysed using techniques such as HPLC–MS or molecular docking simulations. Subsequently, the impact of complex formation on the stability of adhesion proteins on bacterial cell membranes could be explored in greater depth. Additionally, from an industrial application perspective, the retention of antioxidant components and the survival of probiotics under various storage conditions could be further evaluated. Optimization strategies might include improving packaging materials (e.g., light-blocking, nitrogen-flushing) or incorporating natural stabilizers (e.g., gum arabic, pectin) to mitigate phase separation or oxidation processes. In conclusion, the application of Matzhu in food needs to be supported by further fundamental research results.

## Conclusions

This study represents an initial exploration of the effect of the addition of Matzhu during milk fermentation by *L. casei* LC2W. The inclusion of Matzhu during fermentation enhanced probiotic growth in fermented milk. After storage and subsequent in vitro gastrointestinal digestion, Matzhu-supplemented fermented milk exhibited improved antioxidant potential and sustained high probiotic viability. Comprehensive non-targeted metabolomic analysis identified 14 compound classes comprising 1269 differential metabolites, including unsaturated fatty acids, flavonoids, amino acids, and their derivatives. These metabolites were primarily associated with linoleic acid metabolism, amino acid metabolism, flavonoid and flavonol biosynthesis, and ABC transport activity. Consequently, the incorporation of Matzhu facilitated *L. casei* LC2W growth while enhancing the antioxidant activity of fermented dairy products. These promising findings highlight the need for further investigations to elucidate the specific mechanisms by which Matzhu promotes probiotic growth and to explore its prebiotic potential. Moreover, Matzhu supplementation promotes the formation of functional metabolites in fermented dairy products, opening new avenues for the development of dairy formulations possessing beneficial properties. Although this study identified metabolites, we did not further investigate their activities, nor study specific metabolic pathways. Future research should prioritize verifying the antioxidant components and elucidating the precise interactions between these antioxidants and milk components. This would help uncover the potential health benefits of such products and facilitate the integration of Matzhu into fermented dairy applications.

## Supplementary Information

Below is the link to the electronic supplementary material.

Supplementary file 1 (DOCX 24 KB)

## Abbreviations

WSI: Water solubility index
WHC: Water holding capacity
CFU: Colony-forming unit
DPPH, α: α-Diphenyl-β-picrylhydrazyl
ABST: 2,2′-Azino-bis (3-ethylbenzothiazoline-6-sulfonic acid)
LC–MS: Liquid chromatograph mass spectrometer
HMDB: Human metabolome database
PCA: Principal component analysis
OPLS-DA: Orthogonal partial least square discriminant analysis
SD: Standard deviation
ANOVA: Analysis of variance
FC: Fold change
FDR: The false discovery rate-corrected *p* value
QC: Quality control
L0: I.e. L_0_
LZ: I.e. L_1_
