# Supplementary material for: Microbiological, antioxidant and metabolomics changes in fermented dairy products supplemented with Matzhu
Source: Food Sci Anim Resour. 2026 Feb 11;46(1):33. doi: 10.1007/s44463-025-00005-0 (PMC12977300; doi:10.1007/s44463-025-00005-0)
Supplement: Supplementary file 1 — (DOCX 24 KB) [file 44463_2025_5_MOESM1_ESM.docx]

**Appendix A. Supplementary data**

Table A.1 The basic physical and chemical indicators in raw milk.

|  | Fat | Protein | non-fat milk solid | Carbohydrate | Ca | Na |
| --- | --- | --- | --- | --- | --- | --- |
| Milk/100g | 3.80 g | 3.39 g | 9.09 g | 5.31 g | 115 mg | 39.6 mg |

Note: Corresponding methods refer to CFR-Code of Federal Regulations Title 21 Part 131.110 milk in the United States and National Dairy Code Production and Processing Requirements in Canada.

Table A.2 The basic physical and chemical indicators in Matzhu.

| Element | Energy (/100g) | Protein (/100g) | Fat  (/100g) | Carbohydrate (/100g) | Dietary fibre  (/100g) | Na (/100g) | pH  (0.5% w/v) | pH  (1% w/v) | pH  (1.5% w/v) |
| --- | --- | --- | --- | --- | --- | --- | --- | --- | --- |
| Matzhu | 1001 kJ | 12.5 g | 6.9 g | 1.7 g | 63.0 g | 0 mg | 6.54 | 6.41 | 6.32 |

Note: The pH value was measured based on Matzhu dissolved in distilled water (pH 7.0) with thorough mixing.
